# Cross-fostering with up to two surplus piglets relative to sow functional teats: effects on performance and preweaning survival

**DOI:** 10.1007/s11250-026-05250-z

**Published:** 2026-07-28

**Authors:** Rodrigo Dalmina Rech, Bernardo dos Santos Pizzatto, Gabriel Antônio Bona, Pâmela dos Santos Zanatta, Rafael da Rosa Ulguim, Ana Paula Gonçalves Mellagi, Fernando Pandolfo Bortolozzo

**Affiliations:** Department of Animal Medicine, Faculty of Veterinary Medicine, Federal University of Rio Grande do Sul (UFRGS), Porto Alegre, RS Brazil

**Keywords:** Hyperprolificity, Piglet lesion, Piglet loss, Piglet weight, Pre-weaning mortality, Udder lesion

## Abstract

In modern sows, litter size often exceeds the number of functional teats. Therefore, many farms retain surplus piglets within the litter. The present study evaluated the effect of cross-fostering biological litters with up to two surplus piglets relative to functional teats, allowing piglet replacement until day three of lactation. Females were selected (*n* = 201) at farrowing and evenly distributed among treatments: +0 (number of piglets equal to functional teats), + 1 (one surplus piglet), and + 2 (two surplus piglets). Biological litters were formed three to 12 h after farrowing (day 0). Piglets that died or were removed (low viability) during the first three days of life were weighed and replaced. Piglets in the + 2 group tended to have lower immunocrit values than those in the + 0 group (*P* = 0.06). Although the + 2 group exhibited the highest overall piglet loss rate across lactation (*P* ≤ 0.01), litter sizes in the + 1 and + 2 groups were larger than those in the + 0 group (*P* < 0.01). Piglets in the + 2 group also had reduced average daily gain compared with piglets in the + 0 and + 1 groups (*P* < 0.01). Facial and joint injuries were more frequent in the + 1 and + 2 groups on D5 (*P* < 0.01). No differences among treatments were found in udder injury scores, number of functional teats, sow body condition at weaning, daily milk production, or subsequent reproductive performance (*P* ≥ 0.10). Cross-fostering litters with up to two surplus piglets increased the number of piglets weaned without impairing sow performance, although individual piglet growth and early lesions were negatively affected.

## Introduction

Swine prolificacy has improved over the years, increasing the total number of piglets born (Knox 2024). Litter sizes exceeding 16 piglets are now common (Björkman et al. 2017; Hansen 2018; Schild et al. 2020). However, sows typically have 13 to 16 functional teats (Kim et al. 2005; Lundeheim et al. 2013; Earnhardt and Knauer 2019; Obermier et al. 2021), and many litters contain more piglets than there are functional teats available (Oliviero 2023). During the first hours after farrowing, several management strategies are commonly used to address oversized litters and improve piglet survival. These include split suckling, early cross-fostering, the use of nurse sows, and, in some cases, artificial milk supplementation (Baxter et al. 2013). Such interventions aim to ensure adequate colostrum intake and reduce competition for functional teats, which is particularly critical during the first 24 to 72 h postpartum, when most preweaning mortality occurs (Baxter et al. 2013; Panzardi et al. 2013).

The establishment of litters containing surplus piglets during cross-fostering has gained increasing attention in hyperprolific production systems, where the number of piglets born alive often exceeds the number of functional teats (Zanin et al. 2024b; Dos Santos et al. 2025; Pizzatto et al., 2026). However, available information remains limited, and management strategies are not standardized, varying by piglet origin (biological or adopted), number of added piglets, piglet weight, and milk replacer use. Most previous studies have evaluated surplus piglets established within the first day after farrowing. Given that most preweaning mortality occurs during the first three days of life (Panzardi et al. 2013), litters with one surplus piglet may naturally reach a one-to-one piglet-to-teat ratio by day 3 due to early losses (Dos Santos et al. 2025). Therefore, the effectiveness of maintaining surplus piglets under management systems that allow replacement up to day 3 has not been adequately investigated. In this context, the present study evaluates a dynamic management approach that permits piglet replacement during the first three days postpartum, thereby maintaining the intended litter size during this critical early-life period and better reflecting commercial management conditions.

Most previous studies have focused on conditions with a single surplus piglet, leaving limited information on more extreme litter configurations. Higher mortality has not been observed when only one surplus piglet is added (Zanin et al. 2024b; Dos Santos et al. 2025). Litters with two additional piglets, however, show increased preweaning mortality (Vande Pol et al. 2021). Other drawbacks associated with surplus piglets include lesions in both piglets and the sow’s udder (Gallois et al. 2005; Camerlink et al. 2018; Kobek-Kjeldager et al. 2020b; Zanin et al. 2024a, 2024b). Even so, more piglets are weaned per sow, maternal body reserves during lactation appear unaffected, and reproductive performance in the subsequent cycle remains acceptable (Zanin et al. 2024b; Dos Santos et al. 2025).

Therefore, our objective was to evaluate sow and piglet performance in biological litters equalized to contain the same number, or one or two more piglets than the number of functional teats, under a controlled but dynamic management strategy in which piglet viability was monitored and low-viability piglets were removed or replaced until the third day of life. This approach was designed to maintain surplus piglets during the early postnatal period, allowing the assessment of sustained competition for udder access under practical commercial conditions. We hypothesized that cross-fostering with up to two supernumerary piglets may be feasible in hyperprolific herds without impairing sow performance, although potential impacts on piglet survival and welfare should be carefully considered.

## Materials and methods

### Location, housing and animal feeding

The study was conducted on a commercial pig farm with 8,000 females (Camborough, Agroceres PIC, Patos de Minas, MG, Brazil), located in the northern region of Santa Catarina State (26º22’13’’ S, 50º08’40’’ W), Brazil, during the summer and early fall. Environmental conditions were consistent with standard farm management practices, but were not continuously monitored. The sample size was determined by the number of eligible sows available under commercial farm conditions, considering values commonly reported in similar studies and accounting for potential data losses.

At 112 days of gestation, sows were moved to the farrowing rooms and individually housed in crates (2.64 m × 1.48 m). They received a corn–soybean lactation diet: 2 kg/d from housing until farrowing, and *ad libitum* during lactation (3.3 Mcal ME/kg, 18.70% crude protein, and 1.10% digestible lysine), with free access to water.

### Experimental design

Only sows with parity from 2 to 7, 14 or 15 functional teats (Vande Pol et al. 2021), body condition score (BCS) between 2.0 and 3.5 (Young et al. 2003), caliper units from 8 to 15 (Knauer and Baitinger 2015), and ≥ 12 piglets born alive were selected for the study. The selected sows were then evenly distributed among the three treatments according to the number of piglets relative to functional teats (+ 0, + 1, or + 2), while ensuring comparable groups in terms of these traits and the presence or absence of farrowing induction. The + 0 group comprised sows with a number of piglets equal to the number of functional teats (*n* = 68), the + 1 group included one surplus piglet (*n* = 67), and the + 2 group included two surplus piglets (*n* = 66). Litter formation was carried out three to 12 h after farrowing (D0) using biological piglets with vitality scores of three or four (Vande Pol et al. 2021). All piglets were supervised at birth to ensure early access to colostrum, and interventions were performed when necessary to promote successful suckling during the first hours of life. Litter equalization and piglet replacement were conducted only after this initial period, ensuring that all piglets had the opportunity to ingest colostrum prior to any movement. When the number of biological piglets did not meet the treatment requirement, foster piglets with similar weight and age were added, with a maximum of five piglets from no more than two donor sows. At litter equalization, average piglet weights were similar across treatments, and within-litter coefficients of variation (CVs) were kept below 15%. Tail docking, teeth clipping (were performed in accordance with standard farm protocols and national welfare regulations), and iron supplementation were performed within 24 h after birth, and toltrazuril (Cevazuril 5%; Ceva Saúde Animal, Algés, Portugal) was administered on D3. No creep feed was provided during lactation.

All animals were monitored daily throughout lactation. Piglets showing low viability—classified as either emaciated (weak, lethargic, and unable to suckle) or very thin (lethargic but still able to suckle), following (Vande Pol et al. 2021)—were removed from the study and placed with a foster sow. Mortality was recorded daily, and necropsies were performed to determine causes of death, including crushing, starvation, and neurological disorders. Piglets that died or were removed during the first three days of life were weighed and replaced with counterparts of similar age, sex, and weight. Piglet replacement was considered part of the management strategy under evaluation, reflecting practices applied in commercial hyperprolific herds, where early-life interventions are used to maintain litter viability and optimize the use of available functional teats.

### Litter evaluation

Piglets were weighed on D0, D5 and D21 (weaning) using a digital scale with 10 g precision (UR 10000 LIGHT 60/10; Canoas, RS, Brazil). Lesions were assessed at these same time points. Facial and body lesions were scored for severity using a system adapted from Camerlink et al. (2018): 0 = no injuries; 1 = minor lesions not penetrating beyond the dermis or superficial scratches; and 2 = deep lesions penetrating beyond the dermis with multiple scratches. Joint injuries were scored following KilBride et al. (2009): 0 = no lesion; 1 = less than 25% of the joint/dermis injured; 2 = 25–50% injured; and 3 = more than 50% injured.

The immunocrit ratio was measured in a subset of 63 litters, with sampling distributed across treatments during the study period and covering the different phases of data collection. Blood samples were collected between 24 and 36 h after birth. Approximately 5 mL of blood was obtained from the jugular vein using a 25 × 0.8 mm hypodermic needle and syringe and transferred into clot-activator tubes. After coagulation, samples were centrifuged at 1,000 × *g* for 10 min, and 2 mL of serum were aliquoted into Eppendorf microtubes and stored at − 20 °C. For immunocrit analysis, 50 µL of serum was mixed with 50 µL of 40% ammonium sulfate, then centrifuged in a hematocrit microcapillary tube at 12,700 × *g* for 5 min. The length of the precipitated protein column was measured and divided by the total column length to calculate the immunocrit ratio (Vallet et al. 2013).

### Sow evaluation

At farrowing and weaning, caliper measurements (Knauer and Baitinger 2015), BCS (Close and Cole 2001), and backfat thickness (BT) were recorded. BT was measured using a Renco LEAN-MEATER device (Renco, MN, USA) according to the protocol described by Magowan and McCann (2006). Udder lesions were assessed in three regions (anterior, middle, and posterior), and the number of functional teats was counted on D0, D5, and D21. Udder lesions were scored according to Gallois et al. (2005): 0 = no injury; 1 = one or two superficial scratches not penetrating the full dermal thickness; 2 = three or more superficial scratches; 3 = superficial scratches plus fewer than three deep wounds; 4 = superficial scratches plus three or more deep wounds; 5 = large deep lacerations; and 6 = deep and infected wounds. Milk yield was calculated from litter weight and growth rate following the equations from Noblet and Etienne (1989): estimation for D0 to D5 (kilograms/day/piglet) = (2.64 × ADG) + 67; estimation for D0 to D21 (kilograms/day/piglet) = (2.5 × ADG) + (80.2 × BWI) + 7; where ADG = piglet average daily gain (g/d) and BWI = piglet body weight on D0 (kg). Values were multiplied by the number of piglets for each day.

After weaning, sows were housed in gestation crates where estrus detection was performed once daily using a sexually mature boar. When in estrus, sows were inseminated with an intrauterine insemination dose at 24-hour intervals. Subsequently, weaning-to-estrus interval, percentage of females inseminated, farrowing rate, and total born in the next cycle were recorded.

### Statistical analysis

Statistical analyses were performed using Statistical Analysis System (SAS) software, version 9.4 (SAS Institute Inc., Cary, NC), using the generalized linear mixed models (GLIMMIX procedure). Data are presented as least squares means ± standard error of the mean or as model-based percentages, depending on the variable. The models included litter size (+ 0, + 1, +2) as a fixed effect, and the week of sow selection was included as a random effect. Tukey–Kramer adjustment was applied for multiple comparisons within each model, with statistical significance set at *P* ≤ 0.05. A statistical trend was considered when 0.05 < *P* ≤ 0.10. Piglet-level variables were aggregated at the litter level prior to analysis, and the sow and her litter were considered the experimental unit. Female body condition variables on D0 were included as covariates for the corresponding measurements at weaning. To evaluate the success of sows in weaning all piglets after D3, categories were created based on the number of piglets weaned relative to initial litter size: 0, − 1, −2, − 3, or ≤ − 4 piglets. Residuals and model fit were evaluated for all models. For continuous outcomes, residuals were assessed for normality and homogeneity of variance, and variables were analyzed under the assumption of normality when these assumptions were met. Stratification analyses for udder lesion scores, BCS, and weaning success class were performed using ordinal multinomial models (GLIMMIX procedure), with a cumulative logit link function. The percentage of piglets replaced, piglet losses (deaths and removals), causes of death, and piglets with injuries were analyzed assuming a binomial distribution. Farrowing rate was analyzed using a binary distribution. Within-litter CV for piglet weight was analyzed using a beta distribution. Repeated-measures models were used for piglet weight, litter weight, and intra-litter CV, including treatments, time (D0, D5, D21), and their interaction as fixed effects, with repeated observations modeled within the experimental unit. Different covariance structures were tested, and the best-fitting structure was selected based on the Akaike Information Criterion (AIC), with model convergence and parsimony considered.

## Results

No differences were observed among treatments for parity, caliper, BCS, number of functional teats, piglets weaned in the previous lactation, or piglets born alive (*P* ≥ 0.28), indicating that the allocation resulted in comparable groups at baseline (Table 1). The proportion of adopted piglets at the time of cross-fostering differed among treatments (*P* = 0.03), with higher values observed in the + 2 group (+ 0: 14.1 ± 1.1%; +1: 14.8 ± 1.1%; +2: 18.0 ± 1.2%).

**Table 1:** Baseline characteristics of sows at farrowing prior to treatment allocation

*n*	Treatments			P-value
	+ 0	+ 1	+ 2	
	68	67	66	
Parity	4.29 ± 0.20	4.13 ± 0.20	4.15 ± 0.20	0.82
Visual body condition score	2.42 ± 0.05	2.42 ± 0.05	2.45 ± 0.05	0.90
Caliper	9.84 ± 0.21	9.95 ± 0.21	10.21 ± 0.21	0.44
Backfat thickness, mm	10.54 ± 0.44	11.01 ± 0.44	10.54 ± 0.44	0.54
Functional teats, *n*	14.60 ± 0.06	14.61 ± 0.06	14.58 ± 0.06	0.91
Previous weaned piglets, *n*	14.62 ± 0.37	14.69 ± 0.36	14.25 ± 0.36	0.50
Total born, *n*	16.48 ± 0.49	16.51 ± 0.50	17.09 ± 0.51	0.62
Born alive, *n*	15.71 ± 0.27	15.63 ± 0.27	16.20 ± 0.27	0.28

Values are presented as mean ± standard error of the mean

+ 0 group: equal number of piglets and functional teats, + 1 group: one surplus piglet beyond the number of functional teats, + 2 group: two surplus piglets beyond the number of functional teats

Previous weaned piglets: piglets weaned in the previous lactation. Total born: total piglets born in the current farrowing. Born alive: piglets born alive in the current farrowing

### Litter evaluation

Piglets in the + 2 group tended to have lower immunocrit values than those in the + 0 group (difference = 0.015; 95% CI: −0.0003 to 0.0301; adjusted *P* = 0.06), while the + 1 group did not differ from the other treatments (*P* > 0.05; Table 2). Litter sizes on D0 and D5 differed among all treatments (*P* < 0.01) (Table 2). At weaning (D21), litter sizes in the + 1 and + 2 groups were similar, and both exceeded those of the control group (*P* < 0.01). The + 2 group showed a higher piglet replacement rate than the + 1 group up to day 3 post–cross-fostering (*P* = 0.04), with no difference compared to + 0. In addition, the + 2 group had a higher overall loss rate (15.0%) than the + 0 (8.8%) and + 1 (9.6%) groups. Compared to + 0, the odds of loss were greater in the + 2 group (OR = 1.8; 95% CI: 1.32 to 2.56; *P* < 0.01), and a similar increase was observed compared to + 1 (OR = 1.7; 95% CI: 1.22 to 2.31; *P* < 0.01). No difference was observed between the + 0 and + 1 groups (*P* > 0.05). No significant differences in mortality causes were observed among treatments (*P* ≥ 0.15). Although treatment had a significant effect on the piglet removal rate (*P* = 0.04), multiple comparisons showed no differences among groups (*P* > 0.05).

**Table 2:** Number of piglets at cross-fostering (D0), day five (D5), and day 21 (D21); replacements up to day three after farrowing; losses due to mortality (by cause) and removals from birth to weaning (D0–D21); and immunocrit ratio

*n*	Treatments			P-value
	+ 0	+ 1	+ 2	
	68	67	66	
Litter size D0, *n*	14.60 ± 0.06^c^	15.61 ± 0.06^b^	16.58 ± 0.06^a^	< 0.01
Litter size D5, *n*	14.52 ± 0.04^c^	15.46 ± 0.04^b^	16.48 ± 0.04^a^	< 0.01
Litter size D21, *n*	13.73 ± 0.20^b^	14.46 ± 0.20^a^	14.82 ± 0.20^a^	< 0.01
Replacement, %	3.02 ± 0.54^ab^	2.49 ± 0.48^b^	4.48 ± 0.62^a^	0.04
Mortality D0–D21, %	8.39 ± 1.07^b^	9.22 ± 1.13^b^	14.08 ± 1.4^a^	< 0.01
Removal D0–D21, %	0.33 ± 0.20	0.38 ± 0.22	1.07 ± 0.46	0.04
Cause of mortality D0–D21, %				
Crushing	51.01 ± 6.45	42.86 ± 6.34	39.56 ± 5.28	0.15
Starvation	47.83 ± 6.5	55.06 ± 6.39	55.91 ± 5.28	0.24
Nervous signs	1.16 ± 0.81	2.08 ± 2.08	4.53 ± 2.21	0.47
Immunocrit	0.11 ± 0.01^a^	0.10 ± 0.01^ab^	0.09 ± 0.01^b^	0.06

Values are presented as mean ± standard error of the mean

^abc^Different lowercase letters within the same row are different (*P* ≤ 0.05)

+ 0 group: equal number of piglets and functional teats, + 1 group: one surplus piglet beyond the number of functional teats, + 2 group: two surplus piglets beyond the number of functional teats

**Fig. 1:**
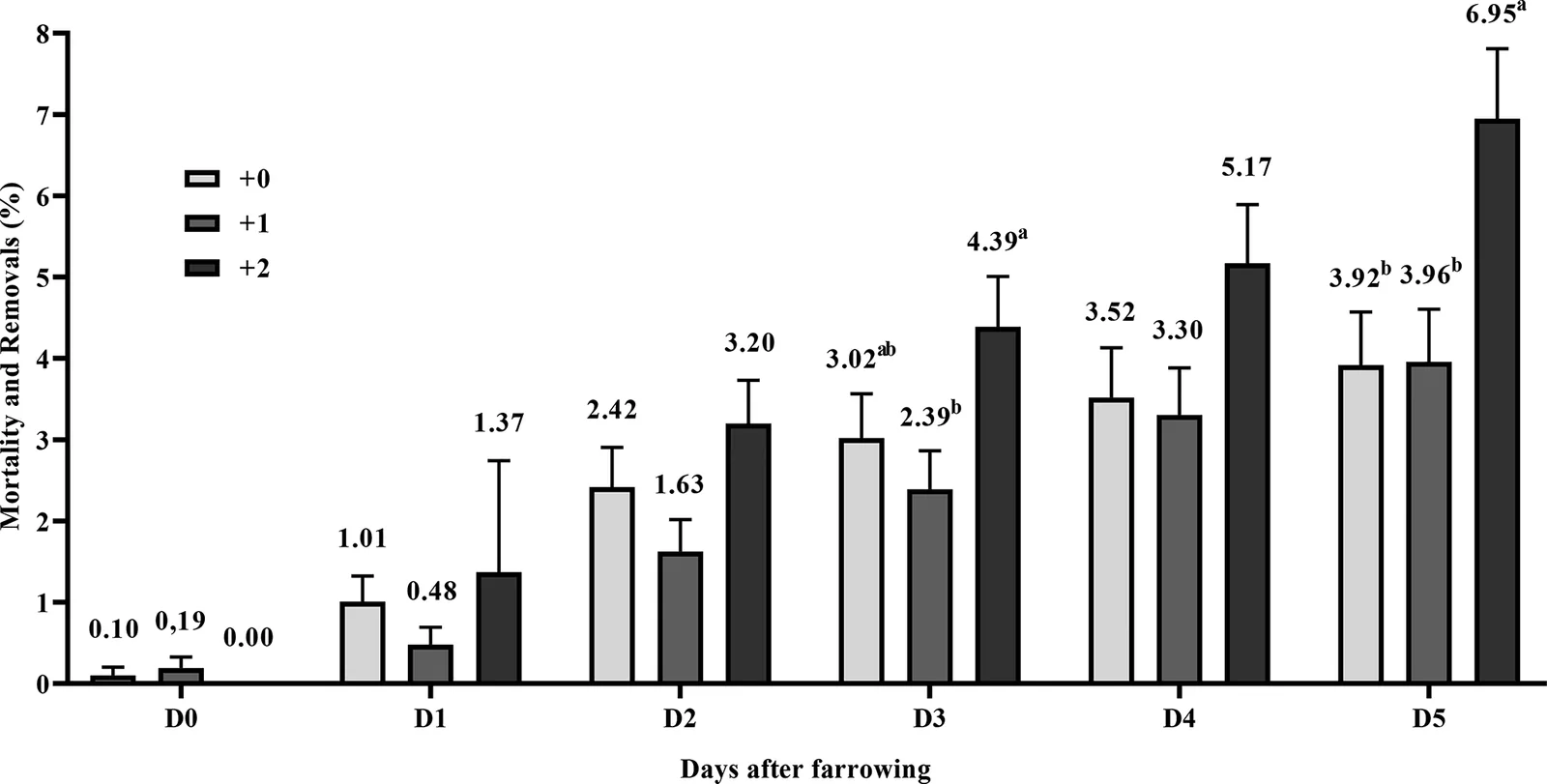
Accumulated daily mortality and removals during the first five days after farrowing. ^ab^At the same time point, values differ statistically (*P* < 0.05). D0: day of farrowing/cross-fostering, + 0 group: equal number of piglets and functional teats, + 1 group: one surplus piglet beyond the number of functional teats, + 2 group: two surplus piglets beyond the number of functional teats. P-value at D3 = 0.03. P-value at D5 < 0.01

**Fig. 2:**
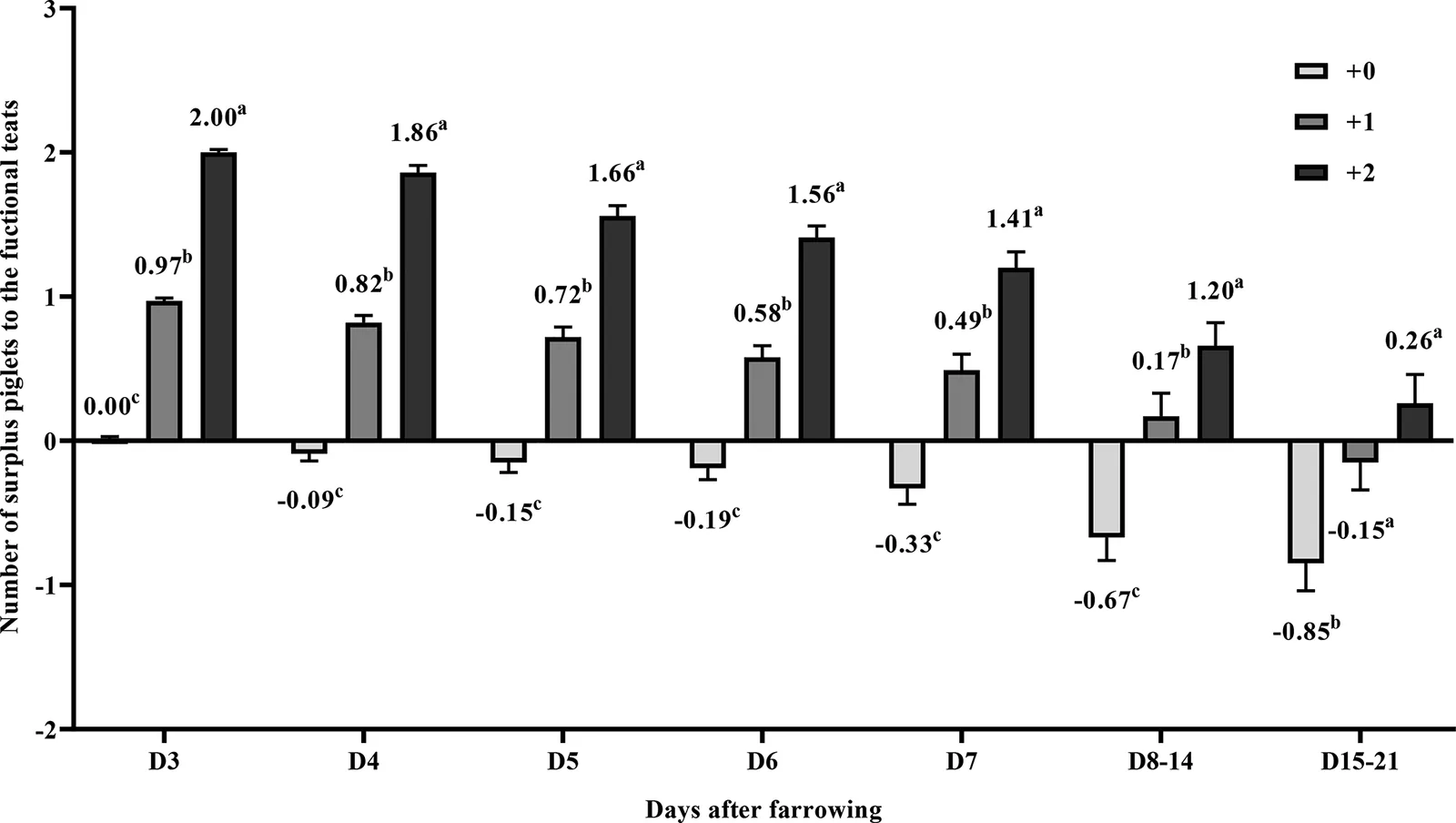
Number of surplus piglets relative to functional teats according to litter size. ^abc^Differ statistically within the same evaluation time (*P* < 0.05). D3: 3 days after cross-fostering and the end of replacements. + 0 group: equal number of piglets and functional teats, + 1 group: one surplus piglet beyond the number of functional teats, + 2 group: two surplus piglets beyond the number of functional teats. P-value at all time points < 0.01

Cumulative mortality and removals up to day five (Fig. 1) were similar between the + 0 and + 1 groups, whereas the + 2 group experienced higher losses (*P* < 0.01). Analysis of surplus piglets relative to functional teats across postpartum days (Fig. 2) showed significant differences among the three groups until day 14 (*P* < 0.01). From days 15 to 21, the + 1 and + 2 groups were similar to each other but remained higher than the + 0 group (*P* < 0.01). Compared to + 0, the + 1 and + 2 groups weaned 0.7 and 1.11 more piglets, respectively.

Piglet growth is summarized in Table 3. No significant interaction between treatment and time was found for litter weight (*P* = 0.15). Litters from the + 1 and + 2 groups were heavier than those from + 0 (*P* < 0.01). A significant treatment × time interaction was observed for individual piglet weight (*P* < 0.01). On D0, piglets in the + 0 group were lighter than those in the + 2 group, but no differences among treatments were detected on D5 and D21. A similar interaction occurred for intra-litter CV. No differences in intra-litter CV were observed among treatments on D0 and D5. However, at weaning (D21), the + 2 group showed greater variability than the + 0 group (difference = 3.51%; 95% CI: 0.26 to 6.76; *P* = 0.02), while the + 1 group showed intermediate values and did not differ from the other treatments (*P* > 0.05). Piglets in the + 2 group had lower ADG during the first five days postpartum (D0–D5) compared to the + 0 and + 1 groups (*P* < 0.01). This difference persisted across the full lactation period (D0–D21), with lower ADG observed in the + 2 group compared to both + 0 (difference = 0.0306 kg/day; 95% CI: 0.0169 to 0.0443; *P* < 0.01) and + 1 (difference = 0.0213 kg/day; 95% CI: 0.0074 to 0.0352; *P* < 0.01). No difference was observed between the + 0 and + 1 groups.

**Table 3:** Litter performance from birth to weaning according to litter size at cross-fostering

*n*	Treatments (TRT)			Mean time	*P*-values		
	+ 0	+ 1	+ 2				
	68	67	66		TRT	Time	TRT×Time
Litter weight, kg					< 0.01	< 0.01	0.15
D0	20.41 ± 0.32	22.89 ± 0.32	24.72 ± 0.32	22.67 ± 0.19^A^			
D5	29.33 ± 0.51	32.24 ± 0.51	32.84 ± 0.51	31.47 ± 0.29^B^			
D21	76.02 ± 1.46	78.26 ± 1.47	77.00 ± 0.51	77.10 ± 0.85^C^			
Mean TRT	41.92 ± 0.66^a^	44.46 ± 0.67^b^	44.85 ± 0.67^b^				
Piglet weight, kg					0.22	< 0.01	< 0.01
D0	1.40 ± 0.02^Aa^	1.47 ± 0.02^Aab^	1.49 ± 0.02^Ab^	1.45 ± 0.01			
D5	2.02 ± 0.03^B^	2.08 ± 0.03^B^	2.00 ± 0.03^B^	2.03 ± 0.02			
D21	5.51 ± 0.08^C^	5.38 ± 0.08^C^	5.20 ± 0.08^C^	5.38 ± 0.04			
Mean TRT	2.98 ± 0.04	2.98 ± 0.04	2.90 ± 0.04				
CV litter weight, %					0.01	< 0.01	< 0.01
D0	16.01 ± 0.48^A^	16.41 ± 0.49^A^	15.83 ± 0.49^A^	16.09 ± 0.38			
D5	20.18 ± 0.64^B^	21.22 ± 0.65^B^	22.55 ± 0.65^B^	21.32 ± 0.45			
D21	21.66 ± 0.79^Ba^	23.44 ± 0.80^Bab^	25.17 ± 0.80^Cb^	23.42 ± 0.52			
Mean TRT	19.29 ± 0.51	20.36 ± 0.52	21.18 ± 0.52				
ADG D0–D5, kg	0.118 ± 0.007^a^	0.118 ± 0.007^a^	0.095 ± 0.007^b^	-	< 0.01	-	-
ADG D0–D21, kg	0.180 ± 0.006^a^	0.170 ± 0.006^a^	0.149 ± 0.006^b^	-	< 0.01	-	-

Values are presented as mean ± standard error of the mean

Comparisons among treatments within each time point (lowercase letters) represent the primary analysis: ^abc^Different lowercase letters within the same row are different (*P* ≤ 0.05); ^ABC^Different uppercase letters within the same column are different (*P* ≤ 0.05)

Mean TRT: overall mean of the treatment, Mean time: overall mean of the time, D0: cross-fostering, ADG: average daily gain

+ 0 group: equal number of piglets and functional teats, + 1 group: one surplus piglet beyond the number of functional teats, + 2 group: two surplus piglets beyond the number of functional teats

Piglet lesion assessments are shown in Table 4. On D5, the frequency of severe facial lesions (score 2) was higher in the + 1 and + 2 groups than in the control group. Compared to + 0, the odds of severe facial lesions were higher in both the + 1 group (OR = 1.44; 95% CI: 1.11 to 1.89; *P* < 0.01) and the + 2 group (OR = 1.33; 95% CI: 1.02 to 1.72; *P* = 0.03), with no difference between the + 1 and + 2 groups (*P* > 0.05). Treatment had no significant effect on body lesions at D5 (*P* ≥ 0.51). The proportion of piglets without joint lesions (score 0) at D5 decreased as the number of surplus piglets increased (*P* < 0.01). Likewise, the proportion of piglets with joint lesion score 2 was greater in the + 1 and + 2 groups than in the + 0 (*P* ≤ 0.01). For the most severe joint lesions (score 3) on D5, the odds were higher in the + 2 group than in both + 0 (OR = 2.00; 95% CI: 1.17 to 3.40; *P* < 0.01) and + 1 (OR = 1.85; 95% CI: 1.11 to 3.08; *P* = 0.01), while no difference was observed between the + 0 and + 1 groups (*P* > 0.05). Despite the higher incidence of facial and joint injuries in the + 1 and + 2 groups at D5, lesion scores for face (*P* ≥ 0.26), body (*P* ≥ 0.18), and joints (*P* ≥ 0.44) were comparable across treatments by weaning (D21).

**Table 4:** Percentage of piglets with different injury scores according to litter size at cross-fostering

*n*	Treatments			P-value
	+ 0	+ 1	+ 2	
	68	67	66	
D5				
Face score 0	67.91 ± 1.49^a^	60.95 ± 1.52^b^	60.74 ± 1.47^b^	< 0.01
Face score 1	14.88 ± 1.13	16.01 ± 1.14	17.59 ± 1.15	0.24
Face score 2	17.21 ± 1.20^b^	23.05 ± 1.31^a^	21.67 ± 1.24^a^	< 0.01
Body score 0	99.19 ± 0.29	99.13 ± 0.29	98.73 ± 0.34	0.51
Body score 1	0.81 ± 0.29	0.87 ± 0.29	0.10 ± 0.30	0.89
Body score 2	0.00 ± 0.00	0.00 ± 0.00	0.27 ± 0.16	0.99
Joint score 0	86.23 ± 1.10^a^	81.68 ± 1.20^b^	77.06 ± 1.27^c^	< 0.01
Joint score 1	7.69 ± 0.85	9.26 ± 0.90	9.99 ± 0.90	0.20
Joint score 2	3.04 ± 0.55^b^	5.79 ± 0.73^a^	7.16 ± 0.78^a^	< 0.01
Joint score 3	3.04 ± 0.55^b^	3.28 ± 0.55^b^	5.89 ± 0.71^a^	< 0.01
D21				
Face score 0	96.90 ± 0.57	97.63 ± 0.49	97.78 ± 0.47	0.43
Face score 1	2.78 ± 0.54	1.75 ± 0.42	1.92 ± 0.44	0.26
Face score 2	0.32 ± 0.18	0.62 ± 0.25	0.30 ± 0.17	0.49
Body score 0	80.24 ± 1.30	82.39 ± 1.22	79.11 ± 1.29	0.18
Body score 1	19.76 ± 1.30	17.61 ± 1.22	20.79 ± 1.29	0.20
Body score 2	0.00 ± 0.00	0.00 ± 0.00	0.10 ± 0.10	1.00
Joint score 0	99.25 ± 0.28	98.66 ± 0.37	98.79 ± 0.35	0.44
Joint score 1	0.43 ± 0.21	0.51 ± 0.23	0.50 ± 0.23	0.95
Joint score 2	0.11 ± 0.11	0.31 ± 0.18	0.10 ± 0.10	0.49
Joint score 3	0.21 ± 0.15	0.51 ± 0.23	0.61 ± 0.25	0.44

Values are presented as mean ± standard error of the mean

^abc^Different lowercase letters within the same row are different (*P* ≤ 0.05)

Degrees of severity: face and body = 0, 1, and 2; joint = 0, 1, 2, and 3

D5: 5 days after farrowing, D21: weaning

+ 0 group: equal number of piglets and functional teats, + 1 group: one surplus piglet beyond the number of functional teats, + 2 group: two surplus piglets beyond the number of functional teats

### Sow evaluation

**Fig. 3:**
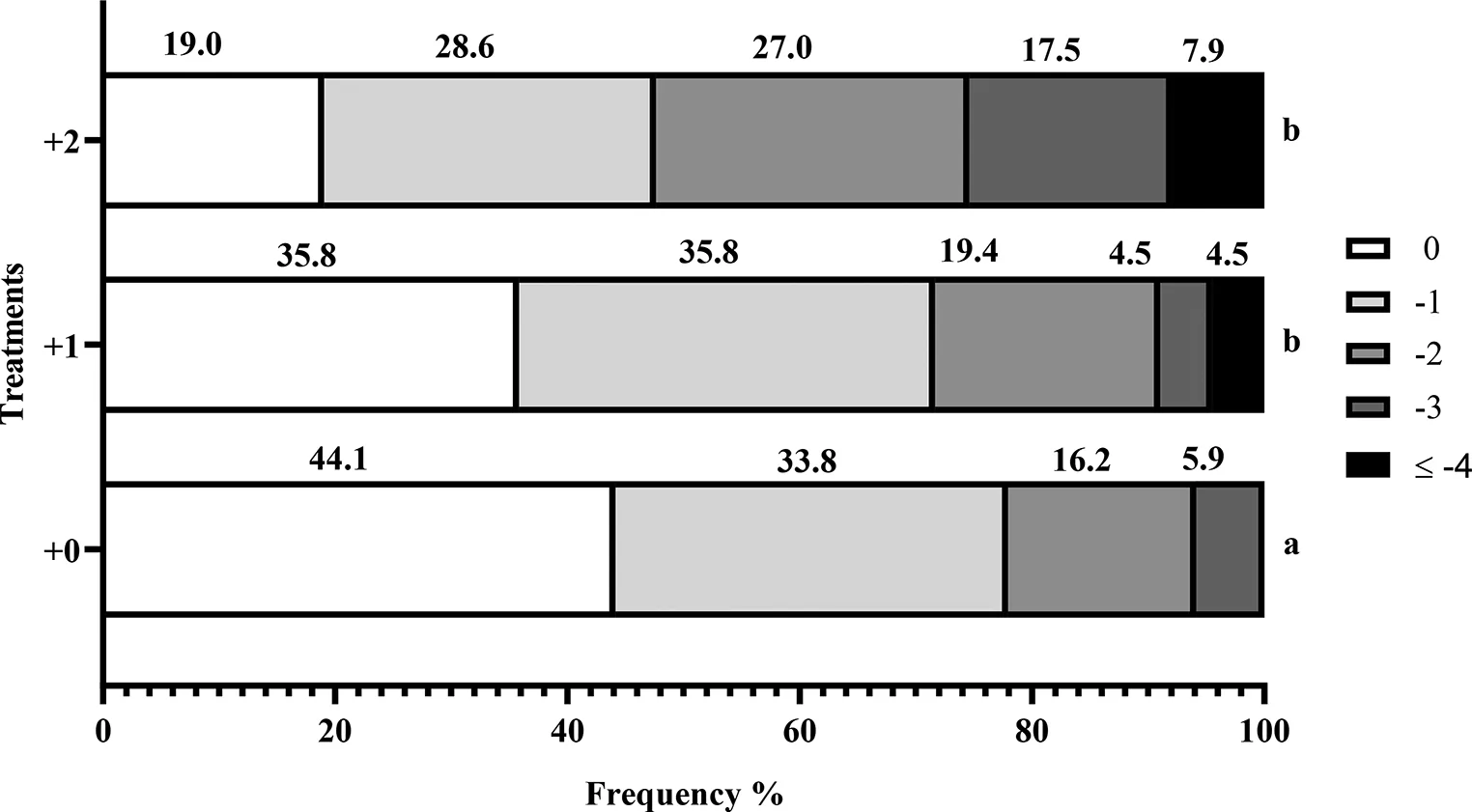
Percentage of sows that weaned the same number of piglets cross-fostered (0), or weaned one (− 1), two (− 2), three (− 3), or four or more ( ≤ − 4) fewer piglets than the initial number, according to litter size at cross-fostering. + 0 group: equal number of piglets and functional teats, + 1 group: one surplus piglet beyond the number of functional teats, + 2 group: two surplus piglets beyond the number of functional teats. ^ab^Groups differ statistically (*P* < 0.05)

No significant differences were found among treatments in any sow injury score or udder region at any time point, nor in the number of functional teats, BCS, caliper, or BT at weaning (*P* ≥ 0.29) (Table 5). Similarly, no treatment effects were observed for body condition change during lactation (*P* ≥ 0.29) (Table 5). Treatment did not influence daily milk production, either during early lactation (D0–D5; *P* = 0.10) or over the full lactation period (D0–D21; *P* = 0.12) (Table 5). The + 0 group showed higher weaning success (*P* < 0.01) than the other groups, with 44.12% of sows maintaining the same number of equalized piglets through weaning (Fig. 3). The + 1 and + 2 groups did not differ, with 35.82% and 19.05% of sows, respectively, weaning the same number of equalized piglets.

**Table 5:** Udder lesions, number of functional teats, daily milk production, and body condition of sows according to treatments

n	Treatments			P-value
	+ 0	+ 1	+ 2	
	68	67	66	
Udder lesions D5, %	34.20 ± 7.33	45.78 ± 7.94	44.71 ± 7.88	0.34
Udder lesions D21, %	67.97 ± 6.60	74.71 ± 6.05	67.68 ± 6.66	0.60
Functional teats D5, n	14.59 ± 0.06	14.57 ± 0.06	14.55 ± 0.06	0.89
Functional teats D21, n	14.38 ± 0.07	14.46 ± 0.07	14.43 ± 0.07	0.63
Milk prod. D0–D5, kg/d	5.52 ± 0.27	5.87 ± 0.27	5.26 ± 0.27	0.10
Milk prod. D0–D21, kg/d	7.85 ± 0.28	8.05 ± 0.28	7.48 ± 0.28	0.12
BCS D21, units	2.76 ± 0.05	2.69 ± 0.06	2.72 ± 0.05	0.59
BCS change D0–D21, units^1^	0.26 ± 0.07	0.15 ± 0.07	0.17 ± 0.07	0.56
Caliper D21, units	10.33 ± 0.34	9.90 ± 0.34	10.32 ± 0.34	0.29
Caliper change D0–D21, units^1^	0.36 ± 0.34	−0.10 ± 0.34	0.28 ± 0.34	0.29
BT D21, mm	9.10 ± 0.34	10.11 ± 0.34	10.19 ± 0.34	0.79
BT change D0–D21, mm^1^	−0.34 ± 0.50	−0.73 ± 0.51	−0.45 ± 0.52	0.71

Values are presented as mean ± standard error of the mean

Udder lesions: percentage of females with at least one lesion in the mammary gland

D0: cross-fostering, Milk prod.: milk production, BCS: visual body condition score, BT: backfat thickness

+ 0 group: equal number of piglets and functional teats, + 1 group: one surplus piglet beyond the number of functional teats, + 2 group: two surplus piglets beyond the number of functional teats

^1^ Body condition change during lactation was calculated as measure at D21 – measure at D0

No significant differences were observed among treatments in subsequent reproductive performance. The percentage of females inseminated was similar across groups (+ 0 = 92.65%, 63/68; +1 = 98.51%, 66/67; +2 = 92.42%, 61/66; *P* = 0.29). Likewise, treatments had no effect on the weaning-to-estrus interval (+ 0 = 4.44 days; +1 = 4.41 days; +2 = 4.98 days; *P* = 0.29). Farrowing rate in the next cycle was unaffected (+ 0 = 88.89%, 56/63; +1 = 96.97%, 64/66; +2 = 88.52%, 54/61; *P* = 0.19), as was the total number of piglets born (+ 0 = 16.45; +1 = 16.44; +2 = 16.85; *P* = 0.75).

## Discussion

In hyperprolific herds, the number of piglets born alive often exceeds the number of teats available. In our study, we evaluated litter equalization strategies involving one or two surplus piglets relative to the number of functional teats. This approach is particularly relevant when the use of nurse sows is limited, and some farms have already adopted such practices despite the scarcity of detailed information. This study evaluated this management strategy under practical conditions, in which piglet demand may exceed milk availability. Previous studies have shown that with one surplus piglet, the number of piglets quickly matches the number of teats within the first few days postpartum (Zanin et al. 2024b; Dos Santos et al. 2025).

In this study, we allowed piglet replacement up to the third day of life, aiming not only to optimize mammary gland utilization but also to examine the impact of surplus piglets during a critical window for piglet mortality—a period that remains understudied. Piglet replacement was considered part of the management strategy, reflecting common commercial practices in hyperprolific herds. However, the dynamic nature of litter composition due to piglet replacement may limit strict causal inference, as changes in litter size and composition during early lactation can influence competition dynamics, teat access, and piglet performance outcomes.

Our results suggest that competition for udder access was similar between the + 0 and + 1 groups but increased in the + 2 group, since piglet replacement and loss rates in the + 1 group were similar to those observed in the + 0 group. In line with these findings, immunocrit values did not differ between these two groups, although a tendency toward lower immunocrit was observed in the + 2 litters. This could have contributed to the higher replacement and mortality/removal rates observed in this treatment. However, immunocrit was assessed in a subset of litters, which may have limited statistical power and should be considered when interpreting these results. Dos Santos et al. (2025) reported that piglets in + 1 litters could take turns during nursing, with most missing only a single nursing session. Although information regarding + 2 litters is lacking, we speculate that they may miss more nursing bouts, which could negatively affect survival. Previous studies have also reported similar preweaning mortality across + 0 and + 1 litters, although a higher removal rate has been observed (Zanin et al. 2024b; Dos Santos et al. 2025). Vande Pol et al. (2021) likewise reported higher mortality in the + 2 group compared to the control. (Kobek-Kjeldager et al. 2020a) found a greater risk of piglet mortality in litters with 2.5 additional piglets, although starvation-related mortality was successfully reduced through the use of milk replacers. These findings indicate that additional management interventions may be needed when increasing the piglet-to-teat ratio in herds intending to use surplus piglets. Notably, despite differences in mortality rates, the causes of death did not differ among treatments, similar to the findings of (Vande Pol et al. 2021). In a factorial study, co-mingling litters alleviated the negative impact of starvation deaths observed in the + 1 group, likely by providing more opportunities for piglets to locate a vacant teat (Zanin et al. 2024b). In our study, the lack of detailed environmental measurements and temporal characterization of mortality may limit the interpretation of some results.

A fast, simple, and cost-effective method for estimating colostrum intake is the immunocrit, which measures total serum protein concentrations at 24 h of age and serves as a proxy for immunoglobulin absorption (Vallet et al. 2013). Low immunocrit values have been linked to impaired growth, delayed puberty onset, and reduced numbers of live-born piglets in subsequent reproductive cycles (Vallet et al. 2015; Vallet and Miles 2017). In our study, piglets from the + 2 group tended to have lower immunocrit values than those from the control group. This trend may help explain the higher mortality observed in the + 2 group, as reduced colostrum intake could have compromised early immune protection and vitality. The inclusion of foster piglets may have introduced additional variability related to colostrum intake, immune status, and early-life adaptation, which should be considered when interpreting the results.

During lactation, both the + 1 and + 2 groups showed improved udder utilization compared with the control, as reflected by the number of surplus piglets maintained throughout the period. Only the + 2 group reached weaning with more piglets than functional teats. This outcome is likely related to allowing piglet replacement up to day three because previous studies that did not permit replacements reported a lower piglet-to-teat ratio and, consequently, the presence of vacant teats during lactation (Zanin et al. 2024b; Dos Santos et al. 2025). In addition, litter size at weaning was greater in the + 1 and + 2 groups than in the control, with increases of 0.77 and 1.11 piglets, respectively, in the present study. Similar results have been reported by studies that formed litters with 1, 2, or 2.5 surplus piglets (Kobek-Kjeldager et al. 2020a; Vande Pol et al. 2021; Zanin et al. 2024b).

Average litter weight remained higher in the + 2 group, with similar individual piglet weights at weaning, although variability was greater than in the + 0 group. Comparable findings were reported by Vande Pol et al. (2021); Zanin et al. (2024b), who observed no significant differences in individual or litter weight at weaning in litters with surplus piglets. These outcomes may reflect limited access to milk and reduced nursing opportunities. Kobek-Kjeldager et al. (2020a), for example, noted improvements in piglet and litter weights when milk replacer was provided in litters with surplus piglets. Charneca et al. (2021, 2023) also documented piglet weight CVs between 21% and 25%, despite using piglets of homogeneous weight during cross-fostering. Increased CV, even in initially uniform litters, has been attributed to variation in milk production and insufficient mammary gland stimulation by piglets (Milligan et al. 2001; Muns et al. 2014). Based on this evidence, we suggest that the higher CV observed in the + 2 group at weaning resulted from suboptimal growth of some piglets due to intense teat competition and reduced access to nursing opportunities. For some variables, statistically significant differences were small and should be interpreted cautiously.

When implementing cross-fostering strategies involving surplus piglets, it is essential to consider animal welfare. In particular, assessment of injuries – especially facial and joint lesions – is a useful indicator because these are closely associated with teat competition (Fraser 1975; Mouttotou et al. 1999; Godyń et al. 2019). Indeed, a higher occurrence of facial abrasion has been reported with increased litter size (Kobek-Kjeldager et al. 2020b). The severity of such injuries may negatively affect piglet development by reducing active behaviors and milk intake, likely due to pain (Mouttotou and Green 1999). In our study, piglets from the + 1 and + 2 groups showed a higher incidence of severe facial and joint lesions on D5. However, these injuries did not persist to weaning, as no significant differences among treatments were observed at that stage. Similarly, Zanin et al. (2024b) reported high low-severity injuries on D5 in cross-fostered litters with one additional piglet, but no differences by weaning. Although piglet competition remained high through the second week because litter size exceeded the number of functional teats (Fig. 2), this pressure lessened during the third week of lactation when more teats became available, reducing competition for nursing. No milk replacer or supplemental feeding was provided, as this reflects the conditions currently applied in commercial farms adopting + 1 and + 2 litter equalization strategies. Under these circumstances, milk intake is likely limited for some piglets, particularly in litters with higher piglet-to-teat ratios. Restricted access to milk may intensify competition, increase lesion occurrence, and impair piglet growth, as observed in the present study. Although the teat order was not evaluated, the patterns observed in lesion scores and growth variability are consistent with increased competition for udder access, suggesting that behavioral dynamics such as teat allocation may play an important role.

Piglet disputes can also cause injuries to the sow mammary system, potentially leading to the permanent loss of gland function (Gallois et al. 2005; Camerlink et al. 2018). With one additional piglet, previous studies have found no effect of litter size on the occurrence of udder lesions (Zanin et al. 2024b; Dos Santos et al. 2025). In our study, even with two surplus piglets, no significant differences were observed among treatments in teat functionality loss or in the severity of udder lesions on either day five or day 21.

Preweaning losses were higher in the + 2 group, where fewer than 20% of females weaned the same number of cross-fostered piglets. By contrast, the + 1 group showed results similar to the + 0 group, suggesting that this strategy may be a feasible management option for hyperprolific herds. From a welfare perspective, the implementation of surplus piglets could be accompanied by management strategies that ensure adequate nutrient intake and reduce competition, such as milk supplementation, optimized cross-fostering practices, and proper colostrum management. Research on the characteristics that predict a female’s success in weaning all piglets is limited. Zanin et al. (2024a) examined factors associated with weaning success and identified parity as an important contributor: younger sows (average parity = 3.2) demonstrated greater potential for successfully weaning larger litters. They also recorded higher milk production on D5 (468.76 g/day/piglet). These findings highlight the need to better characterize females capable of weaning surplus piglets. It is important to note that milk yield in this study was estimated indirectly from piglet growth using equations proposed by Noblet and Etienne (1989), which were developed under specific production conditions that may not fully reflect modern hyperprolific systems. Moreover, under conditions of increased competition for teats, reduced piglet growth may reflect limited access to milk rather than reduced milk production per se. Therefore, milk yield may have been underestimated in treatments with higher piglet-to-teat ratios, and these estimates should be interpreted with caution.

With increased litter size during lactation, greater body condition loss in females is typically expected (Strathe et al. 2017), which may lead to reduced performance in subsequent reproductive cycles (Schenkel et al. 2010). Previous studies have shown no difference—or only slight reductions—in body condition when sows rear litters with one surplus piglet (Zanin et al. 2024b; Dos Santos et al. 2025). In our study, even with two additional piglets, no significant effects of the treatments on body condition or reserve mobilization were observed; consequently, subsequent reproductive performance was also similar across treatments. These findings indicate that the effects of surplus piglets were primarily associated with piglet outcomes rather than sow performance. Factors such as climate, housing design, feeding practices, and herd health status may influence piglet survival and sow performance, and future studies are needed to validate these findings under different production conditions.

## Conclusions

Based on our results, forming a litter with one additional piglet produced outcomes similar to those observed in the + 0 group. However, piglet survival was negatively affected, as the immunocrit ratio tended to decrease when two surplus piglets were added, suggesting reduced passive immunity and potentially greater susceptibility to disease in these animals. Although average daily gain was lower in the + 2 group, piglet and litter weights at weaning did not differ among the three groups. Injuries associated with competition were more common in litters with surplus piglets, but no effects on udder injuries were observed. Sow body condition at weaning and subsequent reproductive performance were not influenced by the addition of surplus piglets. These findings indicate that increasing the piglet-to-teat ratio, particularly with two surplus piglets, may compromise piglet welfare and early-life robustness under the conditions evaluated. Whether additional interventions, such as nutritional supplementation or alternative litter-management strategies, can mitigate these effects should be determined in future studies.

## Data Availability

The data supporting the findings of this study were not deposited in a public repository. They are available from the corresponding author upon reasonable request.

## Declarations

### Declaration of use of AI

During the preparation of this work, the authors used ChatGPT 5.1 (https://chat.openai.com) to perform grammatical checks. After using this tool, the authors reviewed and edited the content as needed, submitted to a professional proof-reading service and take full responsibility for the publication’s content.
